# Atypical Fragility Fractures due to Bony or Soft Tissue Phosphaturic Mesenchymal Tumors: A Report of Two Cases

**DOI:** 10.1155/2023/5614065

**Published:** 2023-04-12

**Authors:** Stephanie M. Clegg, Emily S. Eiel, Sara Fine, Rachel I. Gafni, Mathew J. Most

**Affiliations:** ^1^Department of Orthopedics and Physical Rehabilitation, University of Massachusetts Medical Center, 55 Lake Avenue North, Worcester, MA 01655, USA; ^2^University of Massachusetts Chan Medical School, 55 Lake Avenue North, Worcester, MA 01655, USA; ^3^Department of Orthopaedic Surgery, Hospital of the University of Pennsylvania, 3737 Market Street, Philadelphia, PA 19104, USA; ^4^Department of Pathology, University of Massachusetts Medical Center, 55 Lake Avenue North, Worcester, MA 01655, USA; ^5^National Institute of Dental and Craniofacial Research, National Institutes of Health, 30 Convent Dr. MSC 4320, Bethesda, MD 20892, USA

## Abstract

**Introduction:**

Tumor-induced osteomalacia (TIO) is a rare paraneoplastic disorder where patients present with hypophosphatemia, chronic diffuse bone pain, and occasionally fractures. Benign phosphaturic mesenchymal tumors (PMT) are responsible for the TIO and are largely soft tissue tumors.

**Cases:**

Two male patients with TIO secondary to PMT were reported—one in the bony scapula and the other in the plantar foot soft tissue. The first case describes a 63-year-old Caucasian male, who sustained an intertrochanteric proximal femur stress fracture and approximately two years of diffuse bone pain and hypophosphatemia. Wide excision of a left scapula boney lesion resulted in immediate resolution of his electrolyte abnormalities and bone pain. Case 2 describes a 58-year-old male with four years of multifocal bone pain and atraumatic fractures. A ^68^Ga-DOTATATE-positron emission tomography/computed tomography (PET/CT) scan identified a soft tissue tumor in his plantar foot, which was ultimately excised. He also experienced near immediate resolution of his pain and no additional fractures.

**Conclusion:**

TIO is a rare condition presenting with chronic multifocal bone pain, stress fractures, and hypophosphatemia. These two cases highlight that the causative tumor may originate in soft tissue or bone. Furthermore, a high index of suspicion, along with fibroblast growth factor-23 testing and DOTATATE-PET/CT localization, can help with diagnosis and minimize treatment delays.

## 1. Introduction

Tumor-induced osteomalacia (TIO) is a rare paraneoplastic disorder where typically benign phosphaturic mesenchymal tumors (PMTs) produce excess fibroblast growth factor-23 (FGF23), leading to hypophosphatemia, muscle weakness, and fractures. There are fewer than 1,000 reported cases without sex predilection; the average age of onset is in the 40s; however, pediatric cases are reported. Despite profound and chronic hypophosphatemia, diagnosis is often delayed >2.5 years from symptom onset, with localization of the underlying PMT delaying treatment another five years [1]. We present two cases of TIO due to PMTs—a soft tissue mass in the foot and, to our knowledge, the first reported intraosseous primary PMT of the scapula, as localized by ^68^Ga-DOTATATE-positron emission tomography/computed tomography (PET/CT) scan.

## 2. Case 1

Patient 1 is a 63-year-old man with a history of obesity, sleep apnea, hyperlipidemia, vitamin D deficiency, and stroke, who presented with chronic multifocal bone pain. He underwent uncomplicated left hip arthroplasty for primary osteoarthritis in October 2018. Eight months later, he reported two months of atraumatic right hip pain. Magnetic resonance imaging (MRI) scan showed hyperintense bone marrow edema along the intertrochanteric region and lesser trochanter consistent with a non-displaced intertrochanteric stress fracture, for which he underwent open reduction internal fixation (ORIF). Laboratory results showed mild secondary hyperparathyroidism (parathyroid hormone, PTH 71 pg/mL, normal <64 pg/mL; calcium 8.5 mg/dL) and an elevated alkaline phosphatase (174 U/L, normal 30–115 U/L), thought to be due to fracture.

Six weeks later, the patient was discovered to have a remote history of vitamin D deficiency, and he was initiated on aggressive vitamin D supplementation by his endocrinologist. Secondary hyperparathyroidism resolved; however, bone-specific alkaline phosphatase (BSAP) remained elevated (67.3 *μ*g/L, normal 7.6–14.9 *μ*g), which was attributed to post-operative healing bone. Six months later, rheumatology diagnosed osteopenia, and weekly alendronate were started. A bone scan demonstrated diffuse increased uptake in the proximal humerus, shoulders, distal femur metaphysis, proximal tibial diaphysis, right ankle, thoracic and lumbar spines, sternum, and ribs. He was switched from alendronate to denosumab and underwent five corticosteroid injections into his right hip without significant improvement in pain.

**Figure 1:**
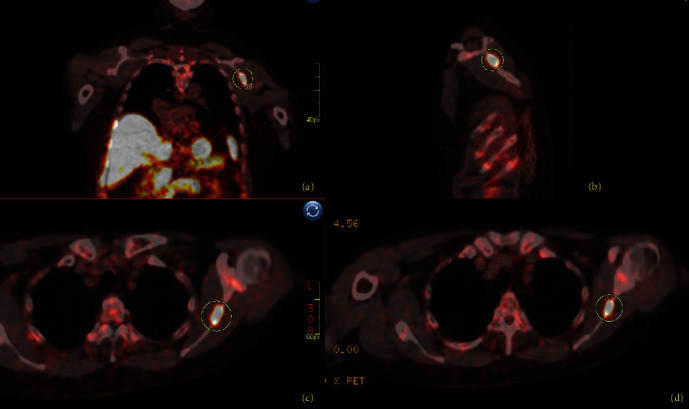
Case 1: left scapula ^68^Ga-DOTATATE-PET/CT images. Coronal (a), sagittal (b), and two axial (c and d) ^68^Ga-DOTATATE-PET/CT images with multiple foci of mild uptake noted with bilateral ribs, costovertebral junctions, and left glenoid concerning for microfractures; and area of hyperintesnse uptake within the left scapula (circle) consistent with primary PMT secreting FGF-23.

**Figure 2:**
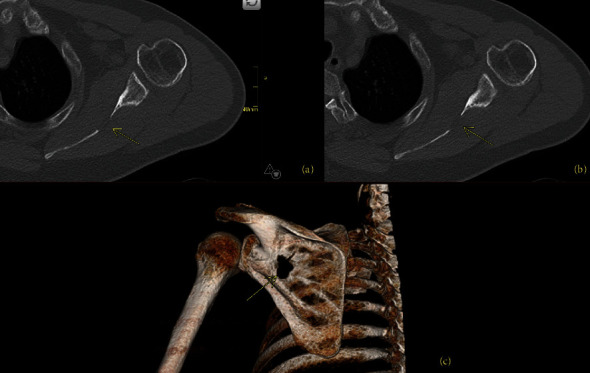
Case 1: left scapula CT and reconstruction images. Axial (a and b) and 3D reconstruction (c) CT images demonstrating the primary bone PMT arising from the left scapular body (arrow) in the patient from Case 1, corresponding to the findings from the DOTATATE-PET/CT in Figure 1.

One year after the ORIF, he had continued multifocal pain including right hip, right thigh, and lumbar spine radiating to the right foot. A lumbar spine MRI demonstrated diffuse bone marrow changes and superior and inferior endplate bowing. He was re-evaluated for a broad differential of bone diseases including hypothyroidism, Paget's disease, hypogonadism, multiple myeloma, and hypercortisolemia. Laboratory results were notable for hypophosphatemia (1.7 mg/dL, normal 2.5–4.5 mg/dL) and an inappropriately low 1,25-OH_2_-vitamin D (12 pg/mL, normal 18–72 pg/mL), suggesting a diagnosis of TIO, which was supported by an elevated FGF23 (403 RU/mL, normal <180 RU/mL). Total-body ^68^Ga-DOTATATE-PET/CT scan identified a lesion in the left scapula (Figure 1), confirmed by CT scan (Figure 2).

**Figure 3:**
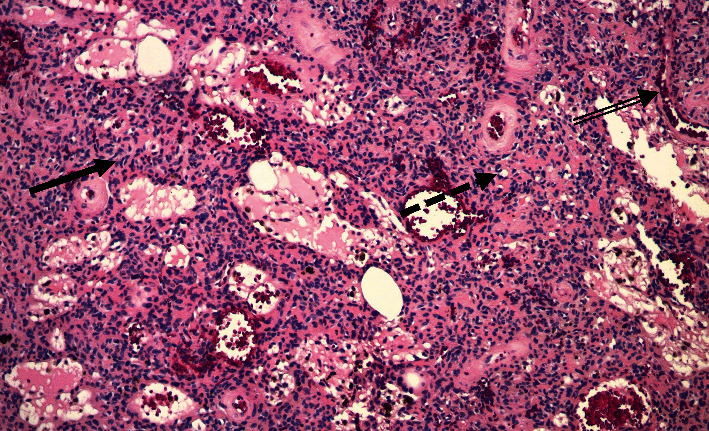
Photomicrograph of the specimen from Case 1. The tumor shows mitotically inactive, cytologically bland spindled to epithelioid cells arranged in a vaguely trabecular pattern (solid arrow) interspersed among hemangiopericytoma-like vessels (double arrow) and microcysts (dashed arrow) (Hematoxylin and eosin, 10×).

Two years after the patient's hip replacement, he underwent wide excision of the left scapular bone lesion, with histology showing bland spindle cells consistent with PMT (Figure 3).

In anticipation of hungry bone syndrome, the patient received aggressive calcium supplementation. On postoperative day 3, hypophosphatemia and hypocalcemia had resolved. Two weeks postoperatively, phosphorus, PTH, calcium, and FGF23 (128 RU/mL) were normal. He reported a subjective increase in the strength of his bilateral lower extremities. Seven months later, he displayed full range of motion and strength of his bilateral upper and lower extremities. One year after surgery, patient continued to feel well without any pain and normal laboratory results except for a mildly elevated BSAP (22.3 *μ*g/L), suggesting ongoing bone remineralization.

## 3. Case 2

Patient 2 is a 58-year-old man with a history of chronic obstructive pulmonary disease (COPD), type 2 diabetes, sleep apnea, testicular cancer, hypertension, and hyperlipidemia, who presented with a four-year history of chronic bone pain in his bilateral hips, back, and ribs, and increasing insomnia, anorexia, fatigue, and depression. In 2016, his primary physician ordered PET and dual energy X-ray absorptiometry (DEXA) scans, which revealed increased activity in the ribs bilaterally and femoral neck osteopenia, respectively. Laboratory results were significant for elevated alkaline phosphatase (251 U/L) and low 25-OH-vitamin D (10.9 ng/mL, normal >20 ng/mL). He was diagnosed with osteoporosis and vitamin D deficiency, for which he was prescribed alendronate and vitamin D supplementation.

Over the next three years, he experienced numerous atraumatic fractures, including left ilium, multiple bilateral lower ribs, right inferior pubic ramus, left sacrum, and multiple cervical and thoracic spinous processes. Laboratory results continued to show elevated alkaline phosphatase (274 U/L) and low 25-OH-vitamin D (10.9 ng/mL). He was referred to an endocrinologist and was found to be hypophosphatemic (1.3 mg/dL) with an elevated PTH (95 pg/mL) and a low 1,25-OH_2_-vitamin D (9 pg/mL). FGF23 was significantly elevated to 354 RU/mL (normal <180 RU/mL), raising suspicion for TIO. Unfortunately, the patient's insurance refused ^68^Ga-DOTATATE scanning for localization, so he was started on medical management with calcium, phosphate, vitamin D, and calcitriol with some lab value fluctuations but without clinical improvement.

**Figure 4:**
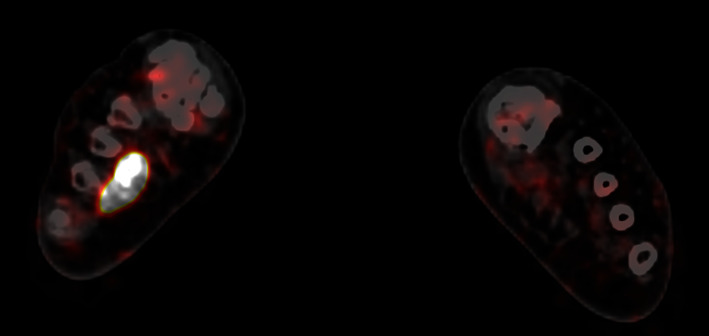
Bilateral feet ^68^Ga-DOTATATE-PET/CT images for Case 2. Axial ^68^Ga-DOTATATE-PET/CT slice through the bilateral feet demonstrating increased uptake in the right foot plantar soft tissue corresponding to the FGF-23 secreting PMT.

**Figure 5:**
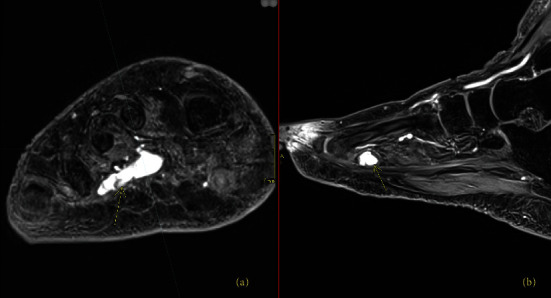
Right foot MRI with contrast for Case 2. Contrast-enhanced axial (a) and sagittal (b) images from an MRI of the foot from patient 2, showing an avidly enhancing soft tissue mass in the plantar aspect of the right midfoot, corresponding to the findings from the DOTATATE-PET/CT in Figure 4.

The patient was ultimately referred to the National Institutes of Health (NIH) in March 2020, where a ^68^Ga-DOTATATE-PET/CT revealed a partially calcified, somatostatin receptor type 2 intensely positive soft tissue lesion in the right plantar foot (Figure 4). MRI showed an enhancing mass in the plantar right midfoot measuring approximately 2.6 cm × 1.4 cm × 1.0 cm (Figure 5).

**Figure 6:**
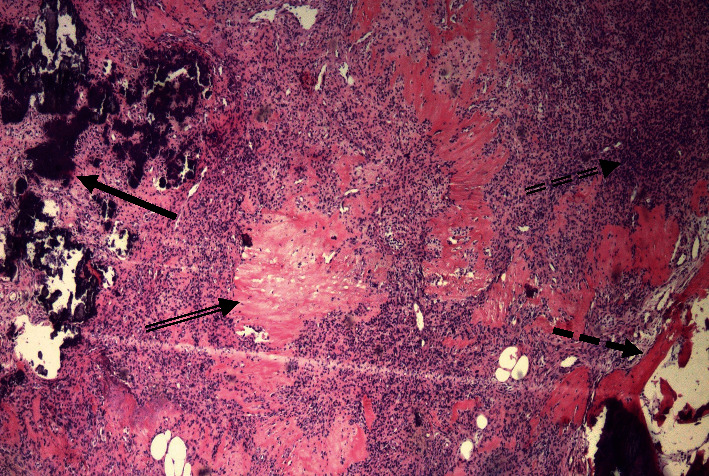
Photomicrograph of the specimen from Case 2. The tumor is composed of spindled to epithelioid cells admixed with few giant cells, with regions of grungy calcification (solid arrow) and hyalinized matrix (double arrow), surrounded by a rim of bone (dashed arrow). The presence of regions with increased cellularity (double dashed arrow) and nuclear atypia are features that have been associated with a more aggressive tumor biology [4] (Hematoxylin and eosin, 4×).

The soft tissue mass was surgically excised, and histopathology confirmed a PMT (Figure 6) with weak staining for smooth muscle actin and fluoroimmunohistochemistry (FISH)-positivity for a fibroblast growth factor receptor-1 (FGFR1) translocation.

Post-operatively, intact FGF23 was undetectable with a rebound in 1,25-OH_2_-vitamin D, signifying complete tumor removal. The patient became hypocalcemic requiring aggressive calcium supplementation, but calcium and phosphate normalized by postoperative day 5 (Table 1).

**Figure 7:**
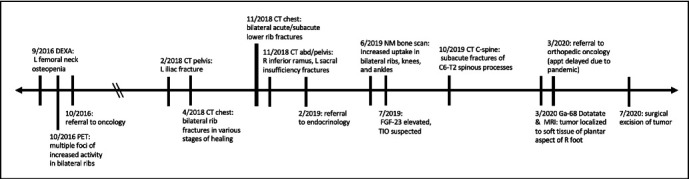
Case 2 timeline. Timeline of the patient's development of TIO symptoms, diagnosis, and treatment for the patient in Case 2.

Three months postoperatively, alkaline phosphatase was still elevated consistent with ongoing skeletal remineralization. Seven months after surgery, the patient reported decreased lesser toe range of motion, but overall felt excellent. One year after surgery, alkaline phosphatase and calcium remained stable and within normal limits. The timeline of this patient's symptoms, workup, and treatment is summarized in Figure 7.

## 4. Discussion

TIO is a rare, acquired cause of excess FGF23, and a bone-derived hormone that regulates blood phosphate levels by promoting renal excretion and suppressing production of active vitamin D (1,25-OH_2_-vitamin D). While acquired FGF23 excess has been rarely reported with malignant tumors such prostate cancer, TIO is usually caused by benign PMTs that are often small and can be anywhere in the body [2, 3]. Soft tissue PMTs most commonly involve the extremities and acral sites, whereas bony PMTs typically involve the appendicular skeleton, cranial bones, and paranasal sinuses [3, 4]. Other bony locations include the distal femur, femoral head, tibial plateau, thoracic and lumbar spines, and sacrum [5]. A recent retrospective review of over 200 patients with PMTs found that 55.8% were in the lower extremities and 29.1% in the head and neck [6]. While there are two reports of periscapular soft tissue tumors causing TIO, we present the first reported case to our knowledge of an intraosseous scapular primary PMT [7, 8]. Of note, PMTs are often remote from locations of reported pain or pathologic fracture, as in our patients. The variable location of PMTs, including bone or soft tissue, emphasizes the importance of whole-body imaging modalities.

As most PMTs express somatostatin receptors, ^68^Ga-DOTATATE-PET/CT is one of the most sensitive and specific modalities to localize PMTs [9]. Unlike simple radiographs, DOTATATE scans can detect extraskeletal lesions, which is crucial as PMTs can arise in both bone and soft tissue. Localization has improved in recent years thanks to improved radionuclide techniques, and decades-long delays in localization are less common [10, 11]. Still, diagnosis and treatment are often quite delayed, with reported average intervals of 2.9–5.7 years from symptom onset to diagnosis and treatment, in part due to the high cost of radionuclide imaging and the lack of recognition of hypophosphatemia, which is not included in most standard chemistry panels [6, 8]. Thus, it is essential that the clinician considers hypophosphatemia in the differential diagnosis of a patient with recurrent fractures and muscle weakness. Time to TIO diagnosis in Case 1 was only 14 months after initial presentation, which is vastly shorter than the reported average. This may be due to the multidisciplinary approach of the work-up, including endocrinology, rheumatology, and orthopedics.

Recent molecular genetics studies have also found that many PMTs express specific translocations, although diagnostic or therapeutic utility is mostly theoretical at this point in time. The *FN1*–*FGFR1* and *FN1*–*FGF1* fusion genes have been estimated to be present in 42–60% and 6% of patients with known PMTs, respectively [12, 13]. While there has been interest in using FISH to diagnose PMTs [12, 14], it is thought there may be too high a risk of false negative results to be used in routine diagnosis. It is also important to note that the tumor must first be localized, which is often the more limiting factor in time to diagnosis. Other recent literature has suggested that these fusion genes may be useful in guiding treatment, such as with FGFR1 inhibitors in patients with unresectable, persistent, or recurrent PMTs [10, 15], but has yet to be studied outside of case reports.

Both patients had persistent bony pain and multiple atraumatic fractures in the setting of chronic vitamin D deficiency and elevated alkaline phosphatase, and were thus misdiagnosed with osteopenia or osteoporosis. However, osteoporosis is four times less common in men than women. Furthermore, these patients were significantly younger than the average age (74–79 years) of rapid bone loss contributing to osteoporosis in men [16]. Therefore, clinicians should have a high index of suspicion for alternative diagnoses, such as TIO in this population, as well as other causes of FGF23-idependent hypophosphatemia, such as primary proximal tubulopathies.

While both patients were older than the average age of TIO onset, their persistent symptoms and lab abnormalities suggested a diagnosis other than osteoporosis [10]. In both cases, discovery of hypophosphatemia led to FGF23 measurement, which prompted whole-body imaging to localize the PMT. Wide margins during surgical resection are essential to induce cure; post-operative FGF23 levels are surrogates for completeness of resection, as demonstrated by almost immediate normalization of FGF23.

## 5. Conclusion

In conclusion, these cases demonstrate the need for a high index of suspicion for TIO in patients with persistent bony pain, pathologic fractures, and lab abnormalities refractory to treatment, especially in patients not typically at risk for osteoporosis. The variable locations of PMTs, including the skeleton and soft tissues, highlight the importance of comprehensive imaging. We recommend FGF23 testing in patients with acquired hypophosphatemia to diagnose TIO, whole-body ^68^Ga-DOTATATE-PET/CT for localization of PMT, and inter-specialty collaboration to achieve timely diagnosis and treatment.

## Figures and Tables

**Table 1:** Pre- and post-operative blood and urine laboratory values for Case 2. Excess FGF23 produced by a PMT promotes renal phosphate excretion and suppresses the production of active vitamin D (1,25-OH_2_-Vitamin D), leading to renal phosphate wasting, hypophosphatemia, elevated alkaline phosphatase, and secondary hyperparathyroidism. The rapid fall in intact FGF23 after tumor removal induces a rebound in 1,25-OH_2_-Vitamin D production and increased calcium requirements as the skeleton begins to remineralize. Concurrently, renal phosphate excretion decreases, and blood phosphate levels return to normal [17].

Laboratory results (normal)	18 weeks pre-operative	DOS pre-operative	POD 0	POD 1	POD 2	POD 3	POD 4	POD 5	12 weeks post-operative	1 year post-operative
Serum Ca (8.7–10.7 mg/dL)	9.1	9.8	9	8.1	7.9	8.2	8.9	9	9.7	9.7
Phosphorus (2.5–4.5 mg/dL)	1.6	1.6	2.1	1.4	1.7	2	2	2.6	3.3	
Urine Ca/Cr (12–244 mg/g Cr)	<1		19	9	<1	<1	11	56		
Urine P/Cr (54–860 mg/g Cr)	<1		644	458	750	863	516	<1		
Alkaline phosphatase (30–115 U/L)	223	177		132					196	76
Vitamin D 1,25-OH_2_ (18–72 pg/mL)	45		29	45	90	142	77	123	89	
PTH (14–64 pg/mL)	127		93	136	217	203	215	50	59	
FGF23 (<180 RU/mL)	502	819		974	563	606	707	598		
Intact FGF23 (<59 pg/mL)			<14	<14	<14	<14	<14	<14		

DOS = day of surgery; POD = post-operative day.

## Data Availability

Access to the data supporting the case reports is restricted due to the Health Insurance Portability and Accountability Act. Deidentified data can be provided by the authors upon request.
